# Impact of the Phoretic Phase on Reproduction and Damage Caused by *Varroa destructor* (Anderson and Trueman) to Its Host, the European Honey Bee (*Apis mellifera* L.)

**DOI:** 10.1371/journal.pone.0153482

**Published:** 2016-04-20

**Authors:** Vincent Piou, Jérémy Tabart, Virginie Urrutia, Jean-Louis Hemptinne, Angélique Vétillard

**Affiliations:** 1 Laboratoire Venins et Activités Biologiques, EA 4357, PRES-Université de Toulouse, Centre Universitaire Jean-Francois Champollion, Albi, France; 2 Laboratoire Evolution et Diversité Biologique, UMR5174, CNRS-Université Toulouse III-ENFA, F-31062, Université Paul Sabatier, Toulouse, France; University of California San Diego, UNITED STATES

## Abstract

*Varroa destructor* is a parasitic mite of the honeybee that causes thousands of colony losses worldwide. The parasite cycle is composed of a phoretic and a reproductive phase. During the former, mites stay on adult bees, mostly on nurses, to feed on hemolymph. During the latter, the parasites enter brood cells and reproduce. We investigated if the type of bees on which *Varroa* stays during the phoretic phase and if the duration of this stay influenced the reproductive success of the parasite and the damage caused to bees. For that purpose, we used an *in vitro* rearing method developed in our laboratory to assess egg laying rate and the presence and number of fully molted daughters. The expression level of two *Varroa* vitellogenin genes (VdVg1 and VdVg2), known to vary throughout reproduction, was also quantified. Results showed that the status of the bees or time spent during the phoretic phase impacts neither reproduction parameters nor the *Varroa* vitellogenin genes levels of expression. However, we correlated these parameters to the gene expression and demonstrated that daughters expressed the vitellogenin genes at lower levels than their mother. Regarding the damage to bees, the data indicated that a longer stay on adult bees during the phoretic phase resulted in more frequent physical deformity in newborn bees. We showed that those mites carry more viral loads of the Deformed Wing Virus and hence trigger more frequently overt infections. This study provides new perspectives towards a better understanding of the *Varroa*-honeybee interactions.

## Introduction

*Varroa destructor* Anderson and Trueman is currently one of the major pests of the European honeybee (*Apis mellifera L*.) and is a threat to the entire beekeeping industry. This mite originally was a parasite of the Asian honeybee (*Apis cerana F*.) and was long mistaken for its sister species *Varroa jacobsoni* Oudemans described at the beginning of the 20^th^ Century. It later shifted host, taking advantage of the developing beekeeping trades worldwide [1–3]; such host shift also occurred in *Varroa jacobsoni* [4].

Once established on *A*. *mellifera*, *V*. *destructor* rapidly spread across Europe and North America, causing huge damage to colonies and thus agricultural losses through a pollination crisis. It reached Europe at the end of the 20^th^ Century and was first described in Czechoslovakia in 1972 [5]. Through its direct parasitic life cycle and the viruses it carries including the Deformed Wing Virus (DWV), implicated in the Colony Collapse Disorder, *V*. *destructor* weakens hives and is one of the main reasons for the 20 to 40% colony loss observed in Northern beekeeping each year [6–8].

Attention was mostly paid to *Varroa’s* biology when it started parasitizing the Western honeybee because of its important role in ecosystem and economy. The mite life cycle consists of two distinct phases: a phoretic phase during which the females stay on adult worker honeybees and feed on hemolymph, and a reproductive phase taking place inside bee brood cells. The second stage starts when phoretic females leave the adult bees and enter a cell containing a 5^th^ larval instar. There they wait until the cell is sealed with wax by worker bees. They then complete reproduction passing through a stereotyped sequence of oviposition, feeding and defecating behaviors [9,10]. The first egg is laid 70 h after entering the cell, is always haploid and will develop into a male. The following eggs, which are laid every 30 h, are diploid and develop into females. Once the eggs hatch, the juveniles develop from protonymphs to deutonymphs before molting into adults. Males mate with the newly molted females, which are brother and sisters in case a single mite entered the cell.

Several studies focusing on the phoretic phase have shown that it seemed to have no aim for the parasite other than providing transport between reproduction sites [11]. Therefore, it could be suppressed without having any visible impact on the mite reproduction in natural conditions [12]. The phoretic mites are more attracted to nurse than forager bees probably because they carry them to their reproduction site [13,14], but the type of bee hosts during the phoretic phase could further influence the mite life cycle by impacting its reproduction. In addition, as the phoretic mites stay on adult bees for a variable amount of time, from one to ten days or more [15], the possible impact of the length of the phoretic phase on nurse bees is of great interest.

The recent development of *in vitro* methods for rearing *V*. *destructor* in laboratory conditions [16–19] provides opportunities to revisit the interactions between bees and *Varroa* in a more controlled environment. Our preliminary *in vitro* rearing, unlike the results obtained by Van Esch and Beetsma [20], suggested that a stay on bees other than nurses could impact the oviposition rate of *Varroa destructor*. The *in vitro* environment also allows new approaches to study the influence of the phoretic phase on damage caused to the host in relation to virus transmission.

Furthermore, the recent advances in the study of *Varroa* gene expression [21–23], especially the two genes of vitellogenin, allow a finer characterization of the parasite reproduction. These two genes are indeed expressed throughout the parasite cycle with a peak of expression 3 days after the cell capping, corresponding to the first oviposition of the female mite [23]. Therefore, they can be employed as markers of the physiological and reproductive status of *Varroa*.

In our study, gelatin cells are used in a new rearing protocol derived from the work of Nazzi and Milani [18], and set up to investigate the impact of both the duration of the phoretic phase and the type of bees on which *Varroa* stays on the parasite reproductive parameters. The expression of the two vitellogenin genes highly related to reproduction is also analyzed, which allowed us to link it directly to different traits of the parasite reproduction. The impact on the bee development is then monitored through the survival and malformations of the developing larvae, and its relation with DWV amounts is studied.

## Materials and Methods

All of our observational and *in vitro* studies were conducted according to European laws for scientific research currently in force. The hives were acquired by the university from local beekeepers aware of the research activities conducted in Albi. The laboratory beekeeping activity and hives were registered (n°81000472) according to the French law and the hives were placed on the university campus under the supervision of qualified authorities.

### Biological material and general *In vitro* rearing procedure

This study was carried out in the South of France in August-September 2014. Eight Buckfast honeybee colonies were maintained on the University campus (INU Champollion, Albi, France). They were left untreated so that the *Varroa* infestation remained high throughout the season. The objective was to rear mites in controlled conditions allowing the performance of our experiments. We used 5.6 mm diameter gelatin capsules (LGA, La Seyne sur Mer, France) following the work of Nazzi and Milani [18]. Holes were pierced in the cap (*ie* the top half) of the gelatin capsule using a thin needle for ventilation. A brood frame was brought back to the laboratory and open capsules were stuck into the cells, not capped yet, of early spinning larvae. The frame was then placed for about 3 h in an incubator (35°C, 60% relative humidity HR) with the top of the brood cells facing the ground, and the larvae slowly slid into the gelatin capsules. One mite was then transferred on each larva less than five hour after the initiation of spinning and the capsules were closed. Prior to their use, the mites were submitted to a phoretic phase in laboratory conditions: 7 days old adult bees previously identified using a water-based marker (Posca™) were taken back from the hive and presented with randomly sampled parasites from a hive brood frame. Host and parasites were kept in experimental cages (Pain type: 10.5 × 7.5 × 11.5 cm; [24]) at 35°C and 60% HR for three days to one week before the transfer of the mites on larvae. Paint spots have been proved to have no observable effect either on the parasite or on the bees themselves [20]. Once the mite had been introduced, the gelatin capsule was immediately closed and these artificial cells were kept in incubators at 35°C and 80% HR [25] until complete bee development (about 11 days). This protocol developed in our laboratory and tested for its reproducibility and relevance was a prerequisite as it defines a controlled environment to study the influence of different factors such as the length and type of bees used during the phoretic phase.

### Phoretic phase influence

To investigate the importance of the phoretic phase on the reproductive success of *Varroa* mites, the general *in vitro* rearing procedure was followed, except that the type of bees and length of the phoretic phase changed. A total of 2,500 emerging honeybees from 3 different hives were identified using a water-based marker (Posca™) one month or one week before the *in vitro* rearing starting date, in order to obtain one month old foragers or one week old nurses [26]. The activity of marked workers was checked before their capture. The foragers were sampled on their way back to the hive and pollen loads were removed to avoid any bias. The nurses were sampled on brood frames and insofar as possible only the bees feeding larvae were selected.

**Fig 1:**
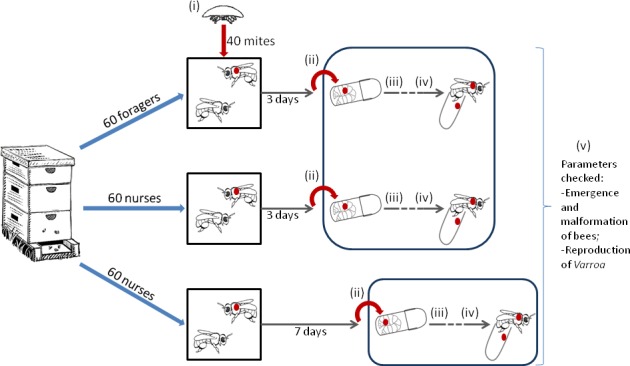
Schematic representation of the experimental design applied to the three hives. Mites experience a different phoretic phase and are then transferred onto spinning larvae to pursue their reproductive cycle in laboratory conditions. Numbers correspond to the moments when mites were sampled to conduct molecular analyses: (i) before the beginning of the phoretic phase; (ii) immediately after the phoretic phase; (iii) three days after the transfer on spinning larvae, at which point the bee is in the last stage of the pharate pupal (or prepupal) period (PP3); (iv) ten days after the transfer on spinning larvae corresponding to the medium pigmented body pupal stage (*pbm*) for the bees; (v) at the emergence of the bee.

The female mites freshly sampled from a brood frame in their reproductive phase–from the spinning larvae stage of the host to medium pigmented body pupae cells–were sorted out into 3 experimental cages corresponding to the 3 treatments: female*s* that spend respectively 3 or 7 days on marked nurse bees before the onset of reproduction, and mites that spend 3 days on forager bees before reproduction. Forty mites allocated to each treatment were kept on 60 marked foragers or nurses in cages (35°C, 60% HR) for 3 or 7 days, according to the treatment, and then transferred on spinning larvae in gelatin capsules less than 5 hours after the initiation of spinning. This experimental design was repeated at two different dates one month apart for each of the 3 hives tested (Fig 1).

*Varroa* mites were allowed to complete their cycle and the gelatin capsules checked after 4 days and at the emergence of the newborn bees. The gelatin cells were searched for eggs, protonymphs, deutonymphs, mature daughters and males. As the amount of *Varroa* mite daughters left after the molecular analysis was not sufficient to dissect the spermatheca, the overall number of potentially fertilized female was estimated based on the survival of the male at the end of the experiment. Malformation, imaginal molt and survival of the bees were also recorded. The fertility (presence/absence of offspring) was analyzed using a generalized linear model (GLM) with the 3 treatments, imaginal molt of the bee, hive of origin and date as explanatory factors. The Akaike information criterion (AIC) minimizing model was retained. The hives and the date of experiment were not considered random because of the too small number of levels in the experimental design [27]. The levels of significant factors were further compared by a χ² test with Bonferroni corrections.

Secondly, the presence and number of fully molted daughters were recorded to assess the success of reproduction. They were analyzed following the same statistical model, using a binomial distribution for the presence/absence of daughter and a Poisson distribution for the number of daughters. Finally, malformation and survival of the emerging bees, both of binomial type dependent variables, were analyzed with a GLM including experimental treatment, emergence of the bee, hive of origin and date as explanatory factors. Significant differences between conditions were compared using a χ² independance test.

### Vitellogenin gene expression and DWV quantification

#### *Varroa* sampling

To quantify the vitellogenin genes expression, the same experimental design as above was applied, except that about 7 mites per treatment and hive were sampled at each of 5 key steps of their development for molecular analyses: (i) before the beginning of the phoretic phase; (ii) immediately after the phoretic phase; (iii) three days after the transfer on spinning larva, at which point the bee is in the last stage of the pharate pupal or prepupal period (PP3); (iv) ten days after the transfer on spinning larvae corresponding to the medium pigmented body pupal stage (pbm) of bees and (v) at the emergence of the bee (Fig 1). The four latter stages were chosen to match with the study of Cabrera Cordon et al. [23]. The daughters found in the last two checks were also sampled in order to analyze and compare their gene expression to the founding females’. They were further subdivided following cuticle pigmentation into older and younger daughters. In case of doubt in the discrimination between mothers and daughters, the mites were not included in the statistical analysis.

All the sampled mites were immediately transferred in a 1.5 ml microcentrifuge tube and ground into 100 μl of RNAlater™ stabilization reagent (Qiagen) using a RNase free pestel (VWR) and stored at -20°C until further analyses. The individuals had to be ground because females *V*. *destructor* stayed at the surface of RNAlater™ [28].

#### Molecular analysis

Total RNA was extracted from the mites sampled as above using the Nucleospin® RNA-mini kit (Macherey Nagel), following the manufacturers’ instructions. RNA from each mite was isolated and the nucleic acid concentrations, along with absorbance ratios A260/A280 were controlled using a NanoDrop 2000 spectrophotometer (Thermoscientific). Forty nanograms of pure isolated RNA were converted into first strand cDNA using the Thermoscript™ RT-PCR system (Invitrogen, Life Technologies). The final volume of 20 μl of total cDNA was kept undiluted at -20°C until performing the quantitative real time PCR (Polymerase Chain Reaction).

The absolute quantification of levels of expression of the *Varroa* targeted genes first required the production of standard curves. The VdVg1 and VdVg2 primers sequences were taken from the study of Cabrera Cordon et al. [23] as well as those of actin, used as a reference gene to normalize vitellogenin transcripts. The primers used to quantify the DWV loads in mites were taken from the study of Chen et al. [29]. PCR were performed as described: denaturation at 95°C for 10 min, 39 cycles as follows: 95°C for 30 s, 59°C for 1 min, 72°C for 1 min and final elongation at 72°C for 5 min using a Labnet MultiGene™ PCR thermal cycler (Sigma, France). The size of the amplicons was checked on a 1% agarose gel electrophoresis. DNA bands were then cut off and purified on a GenElute™ Agarose Spin Column (Sigma) before ligation into the pGEM-T^®^ Vector System (Promega, Lyon, France) and the *Escherichia coli* JM109 (Promega) competent cells were then transformed. Finally, plasmids were isolated using the GenElute™ HP Plasmid Miniprep Kit (Promega) and quantified by spectrophotometry at 260 nm. The standard curves were conceived with serial 10-fold dilutions of the constructed plasmid, ranging from 1×10^1^ to 1×10^9^ copies μL^−1^.

Quantification was performed following the manufacturer’s instructions (Roche Diagnostics) using 1 μl of the pure cDNA sample in a 10 μl reaction mix containing 0.5 μl of both the forward and reverse specific primers. The qPCR were performed in 20 μl capillaries using the LightCycler^®^ 1.5 (Roche Diagnostics), each sample was ran in duplicate. The external standard consisted in amplification of a duplicate of the 1×10^5^ constructed plasmid. The PCR cycles were followed by melting curve analysis according to the manufacturer’s protocol (Roche Diagnostics).

#### Correlation between reproductive parameters, genes expression and malformations

The transcripts levels were analyzed using an ANOVA on the log-transformed data, with the 3 treatments, the 6 mite’s developmental stages, and the hive of origin as explanatory variables. Tukey Honest significance differences tests with Bonferroni corrections were used to compare the levels of significant variables.

The level of gene expression in the two daughters groups (old darkly pigmented daughters and young slightly pigmented daughters) were compared using a Mann-Withney-Wilcoxon test.

GLMs were applied at the PP3 or Pbm stage to correlate respectively fertility or presence of a daughter to the log-transformed VdVg2 and VdVg1 genes expression, hives and phoresia. This allowed the detection of genes as explanatory factors for reproductive parameters. The AIC minimizing model was retained. Both gene expression levels were further compared between reproducing and non reproducing mite groups using Mann-Whitney-Wilcoxon.

Finally, the malformation occurrence in emerging bees, considered as a dependent binomial variable, was also analyzed using a GLM with the 3 treatments and hive of origin as explanatory factors. This model was reiterated on the samples on which DWV levels have been measured, including the viral loads as covariates. The log-transformed DWV loads were also compared between phoretic phases, reproductive stage of the mite and hives, using an ANOVA.

## Results

### Reproduction of the female mites

Eighty-four percent of the mites were fertile and did lay at least one female egg [IC95: 76.2–89.9]. A mean of 1.20±0.12 fully molted daughters per founding mother was counted, but only 0.92±0.11 daughters per mother could be considered as potentially fertile. The presence of living males at the emergence of the bee was variable throughout the hives and often low [46.8% IC95: 37.8–57.9%] (Table 1).

**Table 1:** Survival of the bees and reproductive parameters of the *Varroa*, and comparison with previous *in vitro* or *in naturae* studies. Numbers correspond to frequencies of observation, with the exception of fully molted daughters.

		Bee			Varroa								method and authors	
		newborn molted	newborn alive	Malformation	founding varroa alive	non fertile egg	fertile egg	Protonymph	Deutonymph	mean of fully molted daughter per founding female	Male	daughter frequency	*in naturae* or *in vitro*?	Authors
**Hive 6**	**Nurse_3d**	0.86	0.71	0.29	0.64	-	0.79	0.57	0.36	0.57	0.21	0,36	* *	
	**Forager**	1.00	0.78	0.22	0.67	-	0.67	0.56	0.44	1.00	0.44	0,33		
	**Nurse_7d**	1.00	0.71	0.50	0.86	0.07	0.71	0.71	0.71	1.43	0.43	0,71		
	**Total**	0.95	0.73	0.35	0.73	0.03	0.73	0.62	0.51	1.00	0.35	0,49		
**Hive 7**	**Nurse_3d**	0.80	0.60	0.20	0.93	-	0.87	0.87	0.80	1.87	0.80	0,8		
	**Forager**	0.89	0.83	0.11	0.83	-	0.89	0.72	0.67	1.33	0.44	0,72	*in vitro*	this study
	**Nurse_7d**	1.00	0.72	0.67	0.89	-	0.89	0.89	0.89	1.72	0.56	0,89		
	**Total**	0.90	0.73	0.33	0.88	-	0.88	0.82	0.78	1.63	0.59	0,8		
**Hive 8**	**Nurse_3d**	0.92	0.58	0.17	0.92	-	0.83	0.75	0.58	1.33	0.42	0,58		
	**Forager**	0.94	0.50	0.38	0.81	0.06	0.94	0.75	0.75	1.31	0.38	0,75		
	**Nurse_7d**	0.75	0.63	0.63	0.75	-	0.88	0.88	0.63	0.88	0.50	0,63		
	**Total**	0.89	0.56	0.36	0.83	0.03	0.89	0.78	0.67	1.22	0.42	0,67		
**total**		0.91	0.67	0.34	0.82	0.02	0.84	0,75	0.66	1.32	0.47	0.60		
	** **						0.92			1.26	0.74		*in naturae*	Fuchs and Langenbach [30]
** **	** **						0.83			1.07 (0.83 mated)	0.57		*in vitro*	Donzé et *al*.[10]
**literature**	** **						0.86*			1.45			*in naturae*	Martin [31](*review of 23 european studies)
** **	** **						0.63						*in vitro*	Nazzi and Milani [18]
** **	** **						0.54			0.97	0.46		*in naturae*	Eguaras et al. (1994) from Martin [32]
** **	** **									0.86		0.77	*in naturae*	Ifantidis [33]
** **	** **									1.3			*in naturae*	Schulz [34]

**Fig 2:**
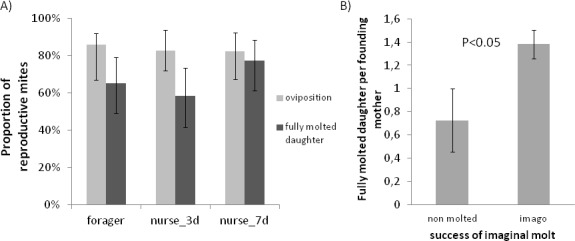
Reproductive parameters of mites in relation to the treatment and emergence success of the bee. A) Oviposition proportion and percentage of cells with at least one mature daughter per foraging condition. Bars show the overall rate ± IC95 (not significant). B) Mean numbers of fully molted daughters in relation to the emergence success of the bee. Mean±SE Non molted (n = 11), imago stage (n = 113).

The fertility was not influenced either by the phoretic treatment, the hive, the date or the imaginal molt of the bee (*i*.*e*. completion of its development until the newborn bee stage) (Fig 2A; GLM, likelihood ratio test, χ^2^ = 4.40, p = 0.48).

The presence or absence of a fully molted daughter at the emergence of the bee is significantly dependent on the hive of origin (GLM, likelihood ratio test, χ^2^ = 9.81, p<0.01) but not on the emergence of the bee, the date or the phoretic condition of the *Varroa* mite (p = 0.26) (Fig 2A).

The number of molted daughters depends on the hive of origin and imaginal molting success of the bee (GLM, likelihood ratio test, hive of origin: χ^2^ = 7.30, p<0.05; imaginal molt of the bee: χ^2^ = 4.31, p<0.05). The molting success positively impacts the number of daughters (0.73±0.27 in cells with an unachieved bee imaginal molt and 1.38±0.12 when the bee reached the emerging adult stage; Fig 2B).

### Vitellogenin gene expression and correlation with reproduction parameters

**Fig 3:**
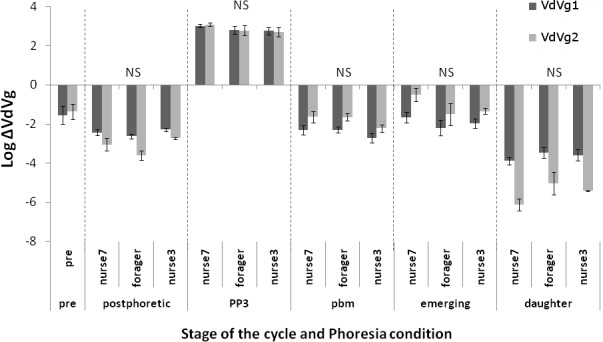
Vitellogenin gene expression from mites in relation with the three treatments. Within stage log-transformed normalized absolute expression of two vitellogenin transcripts of mites that experienced distinct phoretic conditions: mites that spent 3 days on forager bees, mites that stayed 3 days on nurse bees, mites that spent 7 days on nurse bees. The stages of the parasite cycle are: **pre** = prephoretic, mites randomly extracted from brood cells containing different stages of honey bee development and ready to be transferred onto adults; **postphoretic**, mites sampled right after their stay on adult bees in experimental cages; **PP3**, Prepupal stage mites; **pbm**, parasites sampled on brown eyed, medium pigmented thorax honey bee pupae; **emerging**, parasites on the day of honeybee emergence; **daughter**, newly fully molted *Varroa* distinguishable from the mothers. Only the statistical significance for phoretic condition is showed NS = non significant (ANOVA model and post hoc comparisons).

**Fig 4:**
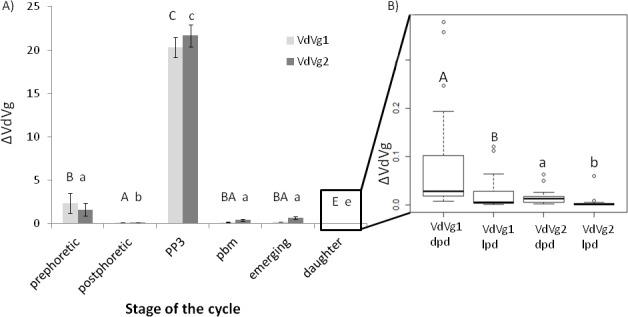
Expression of two vitellogenin genes from mites at different times of the development. A) Normalized absolute expression of the two vitellogenin transcripts at different stages of the parasite cycle, the labels are similar to Fig 3: **prephoretic**, before the transfer onto adults; **postphoretic**, after the stay on adult bees; **PP3**, on prepupal bees; **pbm**, on brown eyed, medium pigmented thorax honey bee pupae; **emerging** mites; **daughter**. Barplot of the mean ± standard error: capital letters indicate the significant differences in the VdVg1 expression, small letters the significance of statistical tests for the VdVg2 expression (ANOVA model and post hoc compaisons p_Bonferroni_<0.003). B) Focus on the normalized absolute expression of the two VdVg transcripts of newly born *Varroa* females after division into two groups according to their age: older daughters (= dpd: darkly pigmented daughters) and younger daughters (= lpd: lightly pigmented daughters). Letters showed the significance of the Mann-Whitney-Wilcoxon test conducted on VdVg1 (capital letter) and VdVg2 (small letter) p_Bonferroni_<0.0083.

The two vitellogenin genes were analyzed independently. Regarding VdVg1, the ANOVA model revealed no interaction between explanatory factors and no effect of the phoretic treatment (p = 0.56) (Fig 3). The hive of origin (p<0.05) and the moment of the *Varroa* cycle (p<0.001) had a significant impact on the gene expression (F_266,250_ = 119.57, p<0.001; Fig 4). Only the expression differences between the emerging mites and the prephoretic, postphoretic or pbm stages, along with the difference between postphoretic mites and pbm stage mites were not significant (Fig 4A). The expression during the PP3 stage (ΔVdVg1 = 20.32±1.17) was the highest and strongly differed from the expression of VdVg1 during the rest of the cycle.

Regarding the VdVg2 expression, the results were very similar to those obtained with VdVg1 (F_266,250_ = 65.31, p<0.001). The ANOVA did not reveal any significant impact of either the phoretic phase or interacting factors (p = 0.24) (Fig 3) but again, the hive of origin (p<0.001) and reproductive time (p<0.001) were highly significant (Fig 4A). Post hoc analyses showed that besides the comparisons between prephoretic, emerging and pbm stage mites, all the pairwise differences were significant (Fig 4A). The level of expression during the PP3 stage of the reproductive cycle was also the highest when compared to the others (ΔVdVg2 = 21.68±1.27).

Daughters have lower levels of expression than their mother at any time of their cycle (ΔVdVg1 = 0.05±0.01 and ΔVdVg2 = 0.02±0.01). Old daughters had significantly higher levels of expressions than their younger sisters (Fig 4B; Mann-Whitney-Wilcoxon VdVg1: W = 568, p<0.001, VdVg2: W = 576, p<0.001).

Interestingly, VdVg2 expression levels (p<0.01), along with the hive (p<0.05), significantly impacted the oviposition success at the PP3 stage. The mites that laid eggs have indeed higher ΔVdVg2 values than the unfertile ones (24.37±1.20 against 14.90±2.64 respectively; Mann-Whitney-Wilcoxon: W = 108, p<0.001). On the contrary, VdVg1 expression is not related to oviposition (Mann-Whitney-Wilcoxon: W = 250, p = 0.21).

At the pbm stage, when trying to link the expression levels to the presence of fully molted daughter, ΔVdVg2 was the only factor retained in the model (GLM, likelihood ratio test, χ^2^ = 8.53, p<0.01). Interestingly, mothers in cells with adult daughters have lower VdVg2 levels than mothers with no daughters (0.24±0.04 against 0.92±0.39 respectively; Mann-Whitney-Wilcoxon: W = 253, p<0.05). Although not involved in the model, VdVg1 showed the same trend (0.10±0.07 when at least one daughter was present, 0.25±0.46 when no adult daughter was observed; Mann-Whitney-Wilcoxon: W = 236, p<0.05).

### Bee survival and malformations

**Fig 5:**
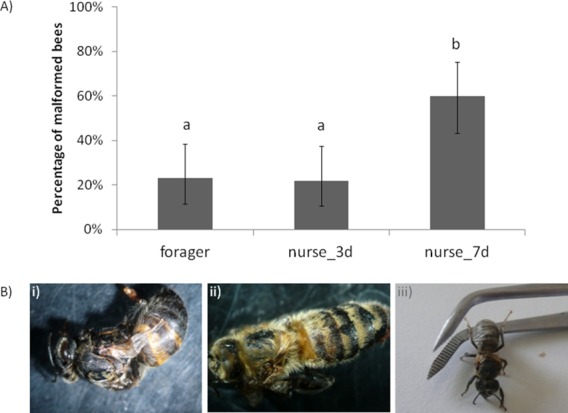
Malformation of bees in relation with the phoretic treatment. A) Proportion of emerging bees with malformations in relation to the phoretic conditions of the *Varroa* they have been parasitized with. Mites that had longer phoretic phase lead to more deformations. Letters shows significant differences analyzed by a GLM model (p<0.001). B) Examples of the most common deformities observed in emerging honeybees: i) atrophy of wings pigmentation anomaly; ii) deformity of wings; iii) atrophy of wings, dark pigmentation.

Interestingly, the only factor impacting the presence of malformations on emerging bees is the phoretic condition the parasites went through (Table 1, Fig 5A, GLM, likelihood ratio test, χ^2^ = 16.42, p<0.001). The *Varroa* mites that stayed on bees for 7 days induced more malformations (χ^2^ nurse_3days *versus* forager: p = 0.88; χ^2^ nurse_7days *versus* forager: p<0.001; χ^2^ nurse_7days *versus* nurse_3days: p<0.001).

Surprisingly, although depending on the presence of malformations (GLM, likelihood ratio test, χ^2^ = 8.11, p<0.001), the survival of the bee at the end of the experiment was not directly impacted by the phoretic phase.

**Fig 6:**
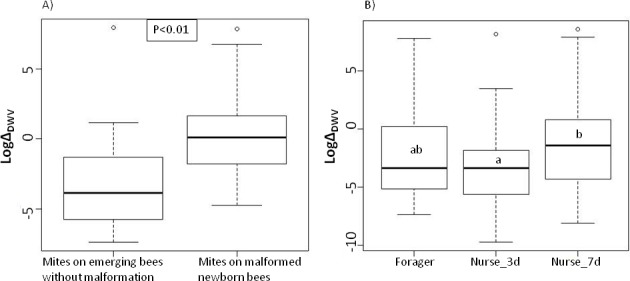
Log-transformed absolute quantification of Deformed Wing Virus (normalized by the expression of Actin). A) In mites sampled on newborn bees in relation to the presence of malformation of the bee (p-value from a Mann Whitney-Wolcoxon test). **B)** In all mites in relation to the previous phoretic experience of these mites. Significant differences from the ANOVA models and post hoc comparisons are indicated with letters.

As the deformity induced by *Varroa* matches the Deformed Wing Virus symptoms (Fig 5B), the levels of DWV were checked in founding female mites at different times of reproduction and could be directly linked to the observation of wing malformations on the newborn bees (Fig 6). The viral load in mites that induced deformity of the emerging bee was significantly higher than in mites coming from perfectly developed bees (Mann-Whitney-Wilcoxon: W = 189, p<0.01). On the contrary, no link between the viral loads and the emergence or death of the newborn bee was found.

The ANOVA on the log transformed viral loads throughout the entire cycle of the mite development resulted in a model retaining the hive of origin (p<0.001), the reproductive time (p<0.001) and the phoretic phase (p<0.01) as significant explanatory factors (F_177,184_ = 7.38, p<0.001; Fig 6). The 7 days spent on nurses led to significantly higher viral loads in mites than the 3 days on nurses (means of log transformed data ± SE: ΔDWV-foragers = -2.33±0.53 ΔDWV-nurse_3d = -3.13±0.40 ΔDWV-nurse_7d = -1.39±0.50). Despite these differences, only a tendency was found between the forager and “7 days on nurses” group and no significant difference between forager and “3 days on nurses” mites. After Bonferroni correction, the reproductive time had only an impact because of the PP3-postphoretic difference (means of log transformed data ± SE: ΔDWV-PP3 = -3.81±0.45 ΔDWV-postphoretic = -1.04±0.48).

## Discussion

### Reproduction of *Varroa*

Our *in vitro* design mimicked the natural conditions experienced by the mite and allowed control of the phoretic phase. The rearing success was validated by the proportion of reproductive mothers and number of fully molted daughters, similar to the measures previously encountered in the literature [18,31,32,35]. The mortality of male is known to be variable and can be high in specific conditions [10,36]. Besides the risk of being squashed by bee larvae [10], we observed that perfectly living males at the pbm (medium pigmented cuticle) or pbd (dark pigmented cuticle) stage of the pupae are frequently found dead one day later, once the bee has reached its imaginal stage.

The age and role of the bees upon which the parasite stays did not impact any of the reproductive traits measured. Neither the percentage of oviposition nor the presence or number of fully molted daughters was affected by the 3 days stay on forager bees, when compared to nurses. This actually corresponds to the results of Van Esch and Beetsma [20] who showed that the *in vitro* stay on foragers, nurses or newly emerged bees did not result in different numbers of offspring (eggs, protonymphs, deutonymphs and adults put together) or in different reproductive female percentage.

We further studied the impact of the phoresia by testing the influence of its length. None of the measured parameters was affected by the two durations tested. Artificial extension of the stay on adult bees has been shown to have no impact on the parasite reproduction in several studies [11,12], except when the phoretic phase is drastically lengthened [37]. Altogether, these results could already suggest that the preferential choice of nurse bees by *Varroa* females [14] is most likely based on the probability of access to a new reproductive site, *i*.*e*. a L5 larva.

Besides the colony of origin, the number of new fully molted females was related to the success of the bee imaginal molt. In cells where the bee died prematurely, most of the protonymphs and deutonymphs could not develop further and died too. Host and parasite cycles are dependent on one another and the completion of the parasite cycle cannot be performed without the completion of its host development [38,39].

### Impact of the phoretic phase on the vitellogenin gene expression and correlation with reproduction parameters

Vitellogenin is a protein playing a key role throughout arthropods life, especially during reproduction [40–45]. As previous studies, we found that the expression of both VdVg1 and VdVg2 in *Varroa* fluctuates throughout its cycle, going through a peak at the prepupal stage when the female lays its first egg [23]. The other periods of the cycle correspond to lower levels of expression. Further arguments in favor of the importance of the vitellogenin genes in the reproductive process of *Varroa* have been highlighted by our paired analysis of the reproductive parameters and genes expression that revealed that reproductive success was indeed correlated to the vitellogenin expression at different stages of the cycle. It could be a further point towards the importance of the *Varroa* cycle synchronization to its host in the parasite reproduction [45–48].

In our study, the phoretic phase had a normalization effect on the mite vitellogenin gene expression since the high variability observed on prephoretic mites (*i*.*e*. random mites sampled at any point of their reproductive cycle) is reduced by the simulated phoretic phase, whether it is 3 or 7 days long.

The qualitative and quantitative changes of the phoresia did not influence any of the two genes studied. However, the levels of vitellogenin transcripts are lower in daughters than in mature adults at any stage of their cycle. Interestingly, it would support the idea that the newly born female mites need a phoretic phase to complete the maturation of their reproductive organs [49]. The fact that the younger slightly pigmented daughters express VdVg1 and VdVg2 at lower levels than their older sisters could indicate that maturation of the mites’ reproductive system is marked by an increasing expression of vitellogenin, probably until it reaches the one of postphoretic females. In such a context, only the first phoretic phase in the parasite life would be essential to the mites. Therefore, it would be interesting to follow the newly born mites Vg gene expression after their transfer on bee nurses and to test which is the required time to become mature. To date, results from several studies including ours have pointed out the subsequent negligible importance of the phoretic phase in the reproduction of mature females [11,12]. At this stage, phoresia would provide an advantageous way of locomotion for the parasite. Because the nurses give access to the brood cells, they are preferentially chosen by the parasites, even though foragers do not prevent them from reproducing.

### Impact on bee development

The malformations observed were mostly the atrophy or deformation of wings, sometimes accompanied by the shortening of the abdomen and pigmentation anomalies (see pictures on Fig 5). Although it was not tested directly on bees, the symptoms described above match those of the Deformed Wing Virus (DWV) disease, one of the most widespread viral diseases in hives, especially when *Varroa* is present [6–8,50–53]. Surprisingly, *Varroa* mites that went through a long phoretic phase induced more frequently these malformations on new adults than short term phoretic parasites, independently of the type of adult bees we tested. As the number of mites at emergence did not impact the development of the pupa, the offspring is probably not as much involved as the single mother in the deformity. In the case of DWV transmission, it has already been shown that the *Varroa* progeny is a negligible vector when compared to mothers [54]. Nordström [55] has showed that wing deformity was strongly correlated to the presence of high loads of DWV and other studies have linked it with the *Varroa*-DWV interaction [8,56,57]. In our study, higher transcripts levels of DWV have indeed been found in mites that did cause wing malformation in bees. Amounts of DWV copies found in mites (10^3^−10^6^ with peaks at 10^8^) were in the same order of magnitude as in the study of Tentcheva et al. [56] but lower than in the work of Gisder et al. [58].

Di Prisco et al. [59] also showed that in weak colonies, a 7 days exposure to *Varroa* resulted in higher viral loads in worker honeybees when compared to 3 days exposed bees. Our data confirmed that the mites spending 7 days on nurses also have higher DWV copies levels than the mites from the two other groups.

Altogether, these results tend to show that *V*. *destructor* induces more wing deformity in emerging honeybees when it has experienced a longer phoretic phase, because of the Deformed Wing Virus transmission. Either because the newly born bee cannot emerge or because it is thrown out of the hive by the worker bees, these malformations often lead to an early death [51,60], as the correlation between deformity and death found in our study suggests. This phenomenon could be intensified in untreated overwintering hives where mites had to go through a long phoretic period because of the absence of brood.

In conclusion, we confirm previous work in that the phoretic phase is not crucial to the reproduction of mature female *Varroa* mites. The phoretic phase is nevertheless important in maturation of the young females and in transport to the reproductive sites. For the first time, the damage to bees has been linked to the length of the phoretic phase, which should be taken into account when managing queenless beehives highly infested with *Varroa* for instance. This research opens new exciting perspectives for the understanding of this host-parasite interaction, in order to later investigate new biological control methods of this pest.
